# COVID-19 pandemic response in Ghana: more to be done

**DOI:** 10.4314/gmj.v55i2s.1

**Published:** 2021-06

**Authors:** David Ofori-Adjei, John Amuasi

**Affiliations:** Editorial Office, Ghana Medical Journal

COVID-19, which emerged in December 2019 and was declared a pandemic in March 2020, has come at a heavy health, economic and social cost to many nations. There have been global efforts to find solutions to both the pandemic and its problems. As a result of unprecedented intense research and development during the pandemic, a better understanding has emerged of the SARS-CoV-2 virus, the disease it causes – COVID-19, its epidemiology, pathophysiology, and clinical management, as well as prevention solutions. However, many questions are yet to be answered, including the role of new variants of the virus in the fight against COVID-19.

The Ghana Medical Journal published a supplement on COVID-19 in December 2020. The supplement provided the Ghana experience in the clinical management of COVID-19 patients. The articles included clinical characterisation, demography, epidemiology, pharmacovigilance, diagnostics, pathophysiological findings, and media reactions to the pandemic in Ghana. In this issue of the journal, we continue highlighting the Ghana experience with an overview of the Ghanaian government's response to the pandemic, mapping of the epidemic, management of surveillance samples, contact tracing, and stigmatisation of SARS-CoV-2 infected individuals.

The pandemic has disturbed the economies of all affected countries, with Ghana's economy growing by a mere 0.4% in 2020 after severe contractions in the second and third quarters.^1^ The pandemic has also badly affected in-person social interactions at family, community, national and international levels. However, each affected country has applied variations of acknowledged mitigation strategies in response to the challenges, including lockdowns and vaccinations. Despite these interventions, several countries have gone through a second and third wave of the pandemic. Though relatively attenuated, African countries have not been spared the recent surges in the pandemic, especially the third wave, which was attributed to the emergence and spread of the Delta variant. The World Health Organisation estimated that the increase in cases was 21% higher than what was seen in the first 48 days of the second wave.^2^ In Ghana, the death rate within the third wave has by far exceeded that in the first and second waves.^3^

Vaccines against COVID-19 have emerged as an important public health intervention to minimise infection and adverse clinical outcomes. It is recognised that vaccination will not entirely prevent viral transmission or infection.

However, having a significant proportion of the population become immune to the virus will ultimately restore economic, social, and academic activities. The benefits of vaccination ultimately depend on how widely and quickly it is conducted, pre-existing seroprevalence levels, and sustaining the gains of appropriate mask-wearing, regular handwashing, and physical distancing.

A major threat to vaccination in low- and middle-income countries is adequate access to the product and appropriate cold-chain infrastructure for the storage, transport and delivery of those vaccines that must be maintained at very low temperatures. Despite continental and regional efforts, vaccination rates are extremely low in Africa, with an estimated just over 1% fully vaccinated.^2^ The vaccination effort has been further affected by supply issues, miscommunication, misinformation and myths, resulting in misunderstanding and mistrust on all aspects of COVID-19, including its existence, propagated especially through social media.^4^ Ultimately, the world's populations desire to see the end of the pandemic. While some things have changed permanently, timely vaccination of the majority of these populations with an effective vaccine will provide the required population immunity needed to abort the pandemic, eliminate the disease, and allow individual, national, and global activities to return to normality as we knew it.

For countries like Ghana, it becomes strategic to enhance clinical, surveillance, and socio-economic data gathering and interpretation for intelligent decision making. COVID-19 has tested the responsiveness and resilience of the healthcare system, and urgent efforts are needed to correct the deficiencies unearthed in Ghana's emergency and regular health services as well as the country's fledgeling biomedical research infrastructure. Above all, it is important for communication on health to be truthful, timely, and in simple and clear language via innovative channels to avoid misunderstanding and miscommunication.

## References

[1] https://www.reuters.com/world/africa/ghanas-economy-grew-04-2020-says-stats-office-2021-04-21/.

[2] World Health Organisation (Africa Office) Africa faces steepest COVID-19 surge yet.

[3] https://www.ghanahealthservice.org/covid19/latest.php.

[4] Kouzy R, Abi Jaoude J, Kraitem A (2020). Coronavirus Goes Viral: Quantifying the COVID-19 Misinformation Epidemic on Twitter. Cureus.

